# The need for education on health related-quality of life

**DOI:** 10.1186/1472-6920-8-2

**Published:** 2008-01-14

**Authors:** Melanie J Calvert, John R Skelton

**Affiliations:** 1 Department of Primary Care and General Practice, University of Birmingham, UK

## Abstract

**Background:**

Health-related quality of life is increasingly recognised as an important outcome measure that complements existing measures of clinical effectiveness. The education available on this subject for different healthcare professionals is varied. This article describes the design, implementation and evaluation of a Special Study Module on Health-Related Quality of Life for undergraduate medical students at the University of Birmingham.

**Methods:**

The course involves 10 hours of "guided discovery learning" covering core concepts of Health-Related Quality of Life assessment including methodological considerations, use in clinical trials, routine practice and in health policy followed by self-directed learning. The taught components aim to provide students with the skills and knowledge to enable them to explore and evaluate the use of quality of life assessments in a particular patient group, or setting, through self-directed learning supported by tutorials.

**Results:**

The use of case studies, recent publications and research, and discussion with a research oncology nurse in task-based learning appeared to provide students with a stimulating environment in which to develop their ideas and was reflected in the diverse range of subjects chosen by students for self-directed study and the positive feedback on the module. Course evaluation and student assessment suggests that quality of life education appears to integrate well within the medical curriculum and allows students to develop and utilise skills of time-management and independent, self-directed learning that can be applied in any context.

**Conclusion:**

We suggest that education and training initiatives in quality of life may improve the quality of studies, and help bridge the gap between research and clinical practice. Resources for curriculum development on health-related quality of life have been developed by the International Society for Quality of Life Research and may prove a useful tool to educators interested in this area.

## Background

Health-related quality of life (HRQoL) is increasingly recognised as an important measure of clinical effectiveness, used to provide evidence for licensing and policy decision-making[1,2]. Yet surveys of clinical investigators have identified a range of problems with the way HRQoL assessment is undertaken or information used, including: lack of understanding of the justification and rationale for HRQoL assessment, lack of guidance on implementing assessments, and limited knowledge of the HRQoL literature[3,4]. These problems highlight the need for continuing medical education on this subject, yet particularly within the undergraduate medical curriculum, educational opportunities are somewhat limited.

A central aim of HRQoL education ought to be an understanding of methodological considerations for HRQoL assessment and how to critically appraise and interpret results. Despite detailed guidance on methodological considerations for implementation and reporting of HRQoL the comparison of treatment effects on HRQoL between two groups is often complicated by missing data and the quality of reporting of many clinical trials assessing HRQoL inadequate[5,6]. This lack of information hampers the appraisal of such trials and brings into question the validity and generalizability of the results.

Courses organised by the European Organization for the Research and Treatment of Cancer and the Eastern Cooperative Oncology Group in response to such findings, and conferences such as those organised by the International Society for Quality of Life Research (ISOQOL), aim to improve the quality of studies and to bridge the gap between HRQoL research and clinical practice[5,7-9]. Such educational initiatives are however primarily aimed at postgraduates, clinical investigators or researchers.

Opportunities for undergraduate students to study this subject in depth appear to be more restricted. For example, at the University of Birmingham the concept of HRQoL is touched on briefly in sessions on chronic illness and palliative care, and students are encouraged to discuss the ethical implications of quality adjusted life years in NHS resource allocation, but have limited formal opportunity to develop a deeper understanding. Special study modules (SSMs) provide an ideal opportunity for undergraduates to study this developing area. Here we report the design and implementation of a HRQoL SSM at the University of Birmingham.

## Methods

### Development of a quality of life SSM

SSMs were introduced into the undergraduate medical curriculum in response to recommendations, made by the General Medical Council in Tomorrow's doctors[10,11], to promote the development of lifelong learning skills and provide an opportunity to explore topics outside the core curriculum. At Birmingham the first in a series of SSMs that form part of the Academic Skills Programme is spread over 8 weeks in the second semester of Year 2 and is a 10 credit module.[12] The 'Quality of Life' course was offered for the first time in the 2003/4 academic year alongside a diverse range of biological and non-biological science subjects including: Cancer of the Gastro Intestinal Tract, Psychotropic Drugs, Drama and Medicine, History of Medicine, and Psychiatry, Law and Ethics. SSMs aim to provide students with an opportunity to study a subject that interests them in greater depth and in doing so to develop and utilise skills of time-management and independent, self-directed learning that can be applied in any context. In addition the HRQoL module aimed to provide students with the skills and knowledge to enable them to explore and evaluate the use of HRQoL assessments in a particular patient group, or setting, and to integrate their findings with other aspects of the medical curriculum including medical ethics and communication skills.

### Course overview

The module consisted of six taught sessions (10 hours in total duration) (Table 1), to provide students with an overview of key areas, followed by self-directed study on the use of HRQoL assessments in area of interest to the student such as: patients with a particular disease, or in a specific group (such as children, or the elderly) supported by tutorials. Sessions were taught and facilitated each year by two members of academic staff. The framework adopted for the taught sessions can be described as "guided discovery learning"[13]. All students were provided with a course handbook that described: the course aims, assessment criteria, evaluation sheets, and a summary for each session containing a brief introduction to the area for study, a series of tasks and a list of key references.

**Table 1:** Overview of the Quality of Life Special Study module sessions and associated learning outcomes

**Session**	**Key Learning outcomes**
**1. **What is QoL and why might we wish to assess it?	• Provide definitions of the terms quality of life and health-related quality of life.
	• Identify situations when the assessment of QoL may be appropriate and provide the rationale for such an assessment.
**2. **How can we measure QoL?	• Identify the key features of generic and disease specific instruments and the advantages/disadvantages of such measures.
**3. **Methodological considerations for the assessment of QoL in a clinical trial.	• Discuss how to choose an appropriate instrument(s) to assess the QoL of patients with a particular disease.
	• Describe methodological issues that should be considered when assessing QoL in a clinical trial.
**4. **Assessing the reporting of QoL in clinical trials	• Be able to evaluate the reporting of QoL in clinical trials.
**5. **Use and assessment of QoL in routine clinical practice	• Explain the potential advantages and disadvantages of routine QoL assessment.
**6. **How is QoL used in licensing/policy making?	• Identify key issues that need to be addressed for QoL to be considered as a credible criterion in the drug regulatory process.
	• Understand the different methods that are used for eliciting patient preferences.
	• Define and perform simple calculations of quality adjusted life years and understand their use in cost-utility analysis.

Each session was structured so that learning outcomes and key concepts were introduced through didactic teaching at the start of the session. These ideas were then reinforced and developed through task-based learning[14]. Examples of tasks that students were asked to undertake to promote self-directed learning included:

• Define HRQoL and using recently published clinical studies identify situations when HRQoL assessment may be appropriate.

• Perform an online search for HRQoL instruments and identify their key properties.

• Plan how HRQoL would be assessed in a clinical trial (based on a case study)

• Evaluate and discuss the reporting of HRQoL in two published studies.

• Find out about clinical assessment of HRQoL (question and answer session with a research oncology nurse).

• Review a presentation and summarise key points on the use of HRQoL in licensing.

• Discuss the use of HRQoL in policy making (based on case studies published by the National Institute for Clinical Excellence).

• Complete a diary that considers how each session relates to the subject chosen for independent study.

Students were encouraged to reflect and conceptualise their findings through facilitated group discussion and to complete a diary considering how each session related to their chosen area for self-directed study. Students were assessed on their contribution to the learning of the group, their understanding and independence in relation to their own study, and on their ability to communicate effectively through a written report and audiovisual presentation.

## Results

The SSM has run with six students each year. Running the course with such a small group of highly motivated students appears to work well. The course evaluation sheets and informal discussion with the students were positive (Table 2) and revealed that they felt that the course complemented other aspects of the curriculum, including concepts covered in their ethics and behavioural science courses such as euthanasia, health care rationing and patient-centred consultation. Students particularly liked the handbooks and task-based learning but felt that supporting material such as original research papers could be provided in a virtual learning environment which we will address for future years. The use of case studies, recent publications and research, and discussion with a research oncology nurse in task-based learning appeared to provide students with a stimulating environment in which to develop their ideas and was reflected in the diverse range of subjects chosen by students for further study. Topics chosen for independent study have included: assessment of HRQoL in people with diseases such as AIDs, arthritis, or post-stroke, or in particular patient groups such as children, or the elderly. Other students have considered diverse topics such as the ethics of HRQoL and euthanasia, whether HRQoL assessments have a place in routine clinical care, and their role in health policy.

**Table 2:** Examples of student feedback

**Were you provided with clearly stated aims and objectives which were met during the course?**	All students stated **yes**
**Did you feel comfortable in contributing to group discussions, if not why not?**	All students stated **yes**, with additional comments stating that "the atmosphere was conducive to contribution" and "made us feel comfortable and at ease"
**Were there any particular strengths or weaknesses in the tutor's approach?**	*Strengths* "Appeared very organised and willing to provide individual time to students" "Organised presentations and handouts.....very helpful" "Encouraged everyone to participate. Having workbook to read before session was useful" "V. approachable. Gave individual attention for essay planning. Good use of email". "Clear instructions. Ensured everyone understood". "Discussion with research nurse was excellent" *Weaknesses * "Sometimes too much paper about trials but it was useful".
**Do you have any suggestions for improvements either in the content or presentation of these sessions or any other comments?**	"Put papers on WebCT" (virtual learning environment) "No, I was very impressed!" "Wide variety of info. Good timetable." "Very well organised and enjoyable. Session with oncology nurse particularly useful as related our knowledge to clinical practice" "Less papers given out".

## Discussion

Formative and summative assessment suggests that quality of life education appears to integrate well within the medical curriculum since some students not only considered the subject in depth but also considered the ethical implications of HRQoL assessment, resource allocation implications, and the potential application of such assessments in facilitating communication and in the promotion of patient centred consultation[15]. It appears therefore to represent an appropriate area for an SSM. It is a fast-developing area of study, though one which is not yet sufficiently central to daily practice to form part of a core curriculum. More interestingly, it offers students an opportunity to work in an area which focuses at one and the same time on key clinical areas, and the evidence which can guide practice: the ethical context of health care: and the relationship between how one makes and how one presents decisions as a health professional. It is therefore very well placed to fulfil the aim of SSMs, to offer learning of generic value.

Whilst SSMs appear to provide an ideal opportunity to introduce undergraduates to the latest research on this subject there is a need for continuing medical education in this area. In recognition of this need the International Society for Quality of Life Research (ISOQOL) has suggested potential topics for inclusion in a HRQoL curriculum and is working to develop a pool of educational materials that could be used when developing courses or workshops on this subject, for undergraduates, postgraduates, clinicians and researchers[9]. Details of topics recommended for consideration when planning an educational program can be found online and should provide educators with a valuable resource[9]. All topics identified by ISOQOL were covered to varying degrees in the SSM; the contents of which are available to educators upon request. During the taught components it was not feasible to cover in detail quality of life issues in specific populations; however, many students chose to develop their knowledge of this in self-directed learning. The student presentations allowed students to benefit from the research undertaken by their peers.

## Conclusion

Evaluation of HRQoL can provide patients, clinicians and policy makers with valuable evidence on the effect of a disease and its treatment on patients' well being and functioning. We suggest that education and training initiatives may improve the quality of studies, and help bridge the gap between research and clinical practice.

## Competing interests

MC is a member of ISOQOL

## Authors' contributions

MC developed the SSM and has participated in the ISOQOL education initiative. JS provided advice on the development of the SSM. Both authors have drafted the manuscript and given final approval for publication.

## Pre-publication history

The pre-publication history for this paper can be accessed here:


